# Men with metastatic prostate cancer carrying a pathogenic germline variant in breast cancer genes: disclosure of genetic test results to relatives

**DOI:** 10.1007/s10689-024-00377-0

**Published:** 2024-05-09

**Authors:** Michiel Vlaming, Margreet G. E. M. Ausems, Gina Schijven, Inge M. van Oort, C. Marleen Kets, Fenne L. Komdeur, Lizet E. van der Kolk, Rogier A. Oldenburg, Rolf H. Sijmons, Lambertus A. L. M. Kiemeney, Eveline M. A. Bleiker

**Affiliations:** 1 Department of Genetics, Division Laboratories, Pharmacy and Biomedical Genetics, University Medical Center Utrecht, Heidelberglaan 100, CX Utrecht, 3584 The Netherlands; 2 Department of Urology, Radboud University Medical Center, Geert Grooteplein Zuid 10, GA Nijmegen, 6525 The Netherlands; 3 Department of Human Genetics, Radboud University Medical Center, Geert Grooteplein Zuid 10, GA Nijmegen, 6525 The Netherlands; 4 Department of Human Genetics, Amsterdam University Medical Centers, Meibergdreef 9, AZ Amsterdam, 1105 The Netherlands; 5 Department of Clinical Genetics, the Netherlands Cancer Institute, Plesmanlaan 121, CX Amsterdam, 1066 The Netherlands; 6 Department of Clinical Genetics, Erasmus Medical Center, Dr. Molewaterplein 40, GD Rotterdam, 3015 The Netherlands; 7 Department of Genetics, University Medical Center Groningen, Hanzeplein 1, GZ Groningen, 9713 The Netherlands; 8 Department for Health Evidence, Radboud University Medical Center, Geert Grooteplein Zuid 10, GA Nijmegen, 6525 The Netherlands; 9 Division of Psychosocial Research and Epidemiology, The Netherlands Cancer Institute, Plesmanlaan 121, CX Amsterdam, 1066 The Netherlands; 10 Department of Clinical Genetics, Leiden University Medical Center, Albinusdreef 2, ZA Leiden, 2333 The Netherlands

**Keywords:** Breast cancer, Family communication, Genetic testing, Metastatic prostate cancer, Psychosocial

## Abstract

Some patients with metastatic prostate cancer carry a pathogenic germline variant (PV) in a gene, that is mainly associated with an increased risk of breast cancer in women. If they test positive for such a PV, prostate cancer patients are encouraged to disclose the genetic test result to relatives who are at risk in case the carrier status changes the relatives’ medical care. Our study aimed to investigate how men who learned they carry a PV in *BRCA1, BRCA2, PALB2, CHEK2* or *ATM* disclosed their carrier status to at-risk relatives and to assess the possible psychological burden for the carrier and their perception of the burden for relatives. In total, 23 men with metastatic prostate cancer carrying a PV completed the IRI questionnaire about family communication; 14 also participated in a semi-structured interview. Patients felt highly confident in discussing the genetic test result with relatives. The diagnosis of prostate cancer was experienced as a burden, whereas being informed about genetic testing results did in most cases not add to this burden. Two patients encountered negative experiences with family communication, as they considered the genetic test result to be more urgent than their relatives. This mixed-methods study shows that metastatic prostate cancer patients with a PV in genes mainly associated with increased risk of breast cancer feel well-equipped to communicate about this predisposition in their families. Carriers felt motivated to disclose their genetic test result to relatives. Most of them indicated that the disclosure was not experienced as a psychological burden.

**Supplementary Information:**

The online version contains supplementary material available at 10.1007/s10689-024-00377-0.

## Introduction

Prostate cancer is the commonest cancer in European, American and African men [1]. Most patients are diagnosed with localized disease but a minority are diagnosed with metastatic disease or develop metastases later on [2, 3]. Between 3 and 19% of patients with metastatic prostate cancer carry a pathogenic germline variant (PV) in a cancer predisposition gene [4–19]. The PVs in high-risk (*BRCA1*, *BRCA2*, *PALB2*) or moderate-risk (*ATM*, *CHEK2*) genes mainly associated with breast cancer in women follow an autosomal dominant inheritance pattern. This means that first-degree relatives have a 50% risk of carrying the same PV and thus may have an increased cancer risk. PVs in these genes may, among others, also be associated with an increased risk of male breast, ovarian, prostate and pancreatic cancer [20–24]. For some of these cancers, periodic monitoring is recommended for proven carriers of a PV (breast cancer screening for women or prostate cancer screening) or carriers may opt for risk-reducing surgery (bilateral mastectomy or salpingo-oophorectomy for women) [25–28]. Patients with castration-resistant metastatic prostate cancer and a PV in one of the *BRCA* genes in germline or tumour DNA may be eligible for treatment with poly-(ADP-ribose) polymerase (PARP) inhibitors [29–31].

### Family communication and cascade screening

Because of the associated cancer risks and the possibility of preventing cancer or detecting it at an early stage, it can be important for relatives to know when a PV is detected in the family. If a PV in a high-risk breast cancer-associated gene is identified in a prostate cancer patient, cascade testing of relatives is advised to inform all relatives at risk, irrespective of their family history [32]. In the Netherlands, relatives of patients with a PV in a moderate-risk gene may also undergo genetic testing, but only if their carrier status changes relatives’ medical care, which makes it slightly different than traditional cascade testing. When applicable, index patients are advised to inform their relatives who are eligible for genetic testing and receive an information letter about the PV to hand out (referred to as the ‘family letter’) [32, 33].

From previous studies, we learned that the majority of prostate cancer patients communicate their genetic test result to at least one relative [34, 35]. Important reasons for communication are to inform relatives about their risk and if applicable to encourage them to have genetic testing done as well. The main reason prostate cancer patients report not informing relatives about their genetic test result is because this result would not change the relatives’ medical care and because of the emotional distance to their relative [34, 35]. In both studies, prostate cancer patients often harboured a variant of unknown significance, a PV in an autosomal recessive gene or in a gene that has very little or no consequences for relatives, which is often different from a PV of known significance in a cancer predisposition gene. Family communication might therefore be less obvious and psychosocial consequences are likely to be different from carriers of a PV in a high-risk or moderate-risk breast cancer gene. When it is known how family communication takes place, this can be used to optimize counselling for metastatic prostate cancer patients and possibly increase the uptake of genetic testing of eligible at-risk relatives.

We aimed to investigate how men who learned they carry a PV in a high-risk or moderate-risk breast cancer gene disclosed their carrier status to relatives at risk. Furthermore, we aimed to explore the possible psychological burden for both the carrier and his relatives, from the carrier’s point of view.

## Methods

This study was part of the DISCOVER study, which has a multicentre, prospective, longitudinal study design [36]. We used a sequential transformative mixed method including questionnaires and interviews to collect our data on family communication about the genetic test result [37].

The study population consisted of patients with metastatic prostate cancer. As part of the DISCOVER study, patients with metastatic prostate cancer were offered genetic counselling and testing by their treating non-genetic healthcare professional (urologist, medical oncologist or nurse practitioner). Consenting patients were at least tested for the cancer susceptibility genes *BRCA1*, *BRCA2*, *ATM*, *CHEK2* and *PALB2*.

Participants in the study were also invited to complete a questionnaire before genetic testing and 4 weeks and 6 months after the genetic test result. The questionnaire could be completed online or with paper and pencil, depending on their preference (see protocol [36]). The study ran from September 2021 to 2023 at 15 hospitals in the Netherlands. Patients received their genetic test results by letter. If a PV was detected, an appointment was scheduled at the genetics department.

In the final questionnaire, patients were asked whether or not they had a PV and if so, whether they wanted to participate in an online interview. The genetic test result was verified at the Genetics, Urology or Oncology department. For patients with a PV in a moderate-risk gene, we verified with the Genetics department whether they had been advised to inform their relatives or not. An invitation letter was sent to patients who consented to an interview, with a proposed date and time. Patients were called for confirmation. Patients were also asked whether or not they wanted a try-out session of the online audiovisual meeting (in Microsoft Teams) with one interviewer (MV) if they were not familiar with the software. Online interviews of 30 to 60 min were held via Microsoft Teams with patients who consented. Two members of the research team (MV and EB) conducted the interview. MV is a male MD and PhD candidate, EB a female professor at the Department of Psychosocial Research with extensive experience in qualitative research and also the supervisor of MV; this was stated at the start of each interview. Each interview started with an explanation of the goal of the interview. It was recorded after patients’ verbal consent and some interviews were automatically transcribed by Microsoft Teams. Field notes were made during the interviews to enhance the interpretation of the interview. We stopped recruitment of patients for the interviews when no new topics emerged in the interviews, indicating data saturation [38]. All interviews took place within 9 months after the patient was informed about the genetic test result.

### Questionnaires and interview

#### Questionnaires

In the first questionnaire, demographic information was collected. A patient’s educational level was determined as low, intermediate or high based on the Dutch Standard Classification of Education [39]. Several topics were addressed in the questionnaire, as described in the protocol of the DISCOVER study [36]. Briefly, the questionnaire consisted of questions about the experience with the offer of genetic testing, the psychosocial impact of genetic testing, the patient’s knowledge of genetic testing, family communication and information about pre-test genetic counselling, not all reported in the current study.

In this paper, we report on the data of family communication about the genetic test result collected with the Informing Relatives Inventory (IRI) [40]. The IRI consists of three domains: knowledge, motivation and self-efficacy. Only the motivation and self-efficacy domains were tested in our study because the items of the knowledge domain were not appropriate or overlapped a lot with the adapted questions from Claes *et al.* that we already used in the DISCOVER questionnaire [41]. The motivation domain is divided in two parts: 13 statements about willingness to inform relatives of the test result (positive motivations) and 17 statements about reasons for not informing relatives (negative motivations). Statements were rated on a 5-point Likert scale indicating whether or not the statement played a role (from ‘no role’ to ‘a large role). Self-efficacy about informing relatives was measured with 7 statements, using a 4-point Likert scale on how sure they were about several statements (from ‘not sure at all’ to ‘very sure’). No cut-off values are available for these domains. In a sample of Dutch patients receiving genetic testing for hereditary breast, ovarian or colon cancer, the scales were reported to be acceptable and reliable and suggested good criterion-related validity [40]. The IRI questionnaire in our study was completed 6 months after the genetic testing results were disclosed to the men with metastatic prostate cancer.

#### Interviews

Interviews were semi-structured and consisted of 12 main questions, divided across seven research topics. The guide was reviewed by an experienced physician (MA). After each interview, the researchers who conducted the interviews checked whether the interview guide needed to be adjusted. The final interview guide is shown in Table 1.

**Table 1:** Interview guide

Research topics	Interview questions
Background information	1. How is your health currently?
Logistics of genetic testing	2. You have been invited by your doctor to have a genetic test for prostate cancer. Who started the conversation about the genetic test? 3. How were you given the results of the genetic test?
Expectations	4. Did you expect to have an inherited predisposition to cancer? If yes, why?
Psychosocial impact	5. You were found to have an inherited predisposition. What was your feeling when you heard this?
Family communication	6 With which of your relatives did you discuss the hereditary predisposition to cancer? 7. How did you feel about discussing this result with your family members? 8. Did you receive a family letter from the Genetics Department to hand out to your relatives? Did it help you? 9. Did you miss any help in discussing the hereditary predisposition with your relatives? What would make discussing the hereditary predisposition easier?
Consequences of test result	10. Are there any relatives who have been tested for the same genetic predisposition? If yes, do you also know what they were told? 11. Are there any relatives who have deliberately not been tested for the same genetic predisposition? If yes, why did they not get themselves tested?
Tips	12. Do you have tips for other men who undergo genetic testing, or for healthcare professionals to improve healthcare?

### Data analyses

Questionnaires: Descriptive statistics were used to determine background variables and to describe the results of the three subscales (positive motivations, negative motivations and self-efficacy) of the IRI questionnaire. IBM SPSS version 29.0 was used.

Interviews: Each interview was transcribed verbatim. For some interviews, a first automatic transcript was made in Microsoft Teams, which was further corrected. A researcher (MV) read through each transcript to make it completely accurate. Transcripts were not returned to the participants, to minimize the participant burden. A coding tree was used in the data analysis process. The data was analysed through an inductive thematic analysis, as described by Braun and Clarke [42] using NVivo, version 12. The thematic analysis consisted of 6 steps: (1) getting familiar with the data, (2) generating initial codes, (3) searching for themes, (4) reviewing the themes, (5) defining and naming the themes and (6) writing the report. All interviews were double coded independently by two researchers (MV and GS) and discrepancies were resolved by discussion. Interview data were compared with the corresponding IRI questionnaire responses for each patient individually. Family communication was assessed as open (all relatives at risk were informed), limited (a selected number of relatives were informed) or closed (no relatives were informed) [35]. The consolidated criteria for reporting qualitative research (COREQ) guidelines were followed and a completed COREQ checklist is provided in the supplements [43].

## Results

In total, 23 patients with a PV completed the final questionnaire and 14 of them consented to an interview. Most patients had a PV in *ATM*, *CHEK2* or *BRCA2*, as shown in Table 2. Patients who consented to be interviewed were significantly more highly educated (p = 0.04) and tended to be younger (p = 0.05) than those who did not.

**Table 2:** Characteristics of patients who did or did not consent to be interviewed at the moment the questionnaire was completed

	Patients with pathogenic germline variant (n = 23)	Consent for interview (n = 14)	No consent for interview (n = 9)	p value
Age in years, median (range)	69 (59 – 82)	67 (69 – 81)	75 (67 – 82)	NS (0.05)
Pathogenic germline variant (n)				NS (0.69)
* ATM*	10	5	5	
* BRCA1*	0	0	0	
* BRCA2*	5	3	2	
* CHEK2*	7	5	2	
* PALB2*	1	1	0	
Time since prostate cancer diagnosis in years, median (range)	2 (0 – 13)	2 (0 – 11)	2 (0 – 13)	NS (0.46)
Number of siblings, median (range)	2 (1 – 12)	2 (1 – 10)	3 (2 – 12)	NS (0.22)
Number of children, median (range)	2 (0 – 4)	1 (0 – 4)	2 (1 – 2)	NS (0.15)
Educational level (n)				0.04
Low	6	2	4	
Intermediate	7	3	4	
High	10	9	1	
Moderate risk cancer genes: advised by genetic department to inform relatives? (n)				NS (0.68)
Yes	13	8	5	
No	4	2	2	

*NS* not significant

## Informing Relatives Inventory

In Table 3, the role that positive and negative motivations play in family communication on the genetic test result are shown as median scores, as well as their self-efficacy to communicate this result.

**Table 3:** Median scores per question on IRI questionnaire (n = 23)

	Median score (range)
Positive motivation	
a. I feel obliged to provide information	4 (1–5)
b. I was encouraged by the health professional	2 (1–5)
c. I already promised my relatives to inform them before I got the result	3 (1–5)
d. I’m encouraged by other relatives to disclose the information	2 (1–5)
e. I think the information can help them to make medical decisions	4 (1–5)
f. A relative asked me about the test result	2 (1–5)
g. I would like to have my relatives’ advice when I make medical decisions	2 (1–4)
h. I need emotional support from my family	3 (1–5)
i. I would like to encourage my relative to go for genetic services	3 (1–5)
j. I would like to encourage my relative to go for regular screening	4 (1–5)
k. I understand the test result well	4 (2–5)
l. I have a close relationship with some of my relatives	4 (1–5)
m. I think relatives’ children should know the information	4 (1–5)
Negative motivation*	
a. I do not have any contact with some of my relatives	3 (1–5)
b. I do not have a good relationship with some of my relatives**	1.5 (1–4)
c. I do not believe the information would be useful to them	3 (1–4)
d. I do not think the relative can handle the information emotionally	1 (1–4)
e. I am worried about being responsible for causing problems in a relationship or marriage	1 (1–2)
f. My relative told me they did not want to know	1 (1–2)
g. I do not have time to tell my relatives	1 (1–2)
h. It is difficult to reach some of my relatives	2 (1–5)
i. I do not want to burden my relative, since he/she is having difficulties	1 (1–2)
j. I consider my relative too young to inform	1 (1–5)
k. It is emotionally difficult for me to share the information	1 (1–3)
l. The information is so complex, I do not know how to share the information	1 (1–2)
m. I do not know who has an increased risk for having hereditary cancer	1 (1–2)
n. Some relatives do not understand how the information applies to them	1 (1–3)
o. I do not want to upset my relatives	2 (1–4)
p. I feel guilty or anxious about the test result	1 (1–2)
q. I feel the information is too personal to share	1 (1–2)
Self-efficacy	
*If you would like to inform your family, how sure are you that you …* a. Find the time to speak them	3 (2–4)
b. Contact them	3 (1–4)
c. Disclose the information clearly	3 (1–4)
d. Cause undesirable turbulence	3 (1–4)
e. Explain what the importance of the information is for them	3 (1–4)
f. Avoid problems in the relationship	3 (1–4)
g. Have enough knowledge	3 (1–4)

Positive and negative motivation: ‘The motivation plays’ 1 = ‘No role’ to 5 = ‘A large role’. Self-efficacy: 1 = ‘Not sure at all’ to 4 = ‘Very sure’.

*n = 9

**n = 8

A detailed score per question in the IRI is shown in Table S1. Mean total scores for the IRI subscales are shown in Table S2. Patients who received advice from the genetics department to inform their relatives scored significantly higher overall on the positive motivation subscale (p = 0.04) and showed no significant differences for the other subscales (p = 0.33, p = 0.25) compared to patients who were not given this advice.

Four themes emerged from the interviews: (1) linking genetic testing to family life, (2) dealing with the germline genetic testing result, (3) navigating family factors in disclosure of genetic testing results and recommendations for cascade testing and (4) evaluating and taking action after disclosure of testing results.

## Theme 1: Linking genetic testing to family life

Reasons for undergoing genetic testing were to possibly opt for an additional treatment option (Pt3, Pt4, Pt7), to detect whether relatives were at risk, and if so, whether relatives could undergo preventive measures (Pt9, Pt11), to contribute to science (Pt3) or out of curiosity (Pt10). Two patients who had genetic testing done to opt for additional treatment options had multiple brothers and no sisters (Pt3, Pt4), of whom one had daughters (Pt4) and the other had no children (Pt3). Pt7 had one sister and no children.

Some patients expected that they had a genetic predisposition (Pt1, Pt3, Pt4, Pt11), two of them (Pt3, Pt11) because of prostate cancer in their family and two (Pt1, Pt4) for unspecified reasons.

*‘My father died of prostate cancer. So I thought, well, maybe that’s a hereditary variant’ (P3)*

Several other patients did not expect to have a genetic predisposition (Pt2, Pt5, Pt7, Pt10, Pt12, Pt13, Pt14), although Pt10 had two relevant cancer cases in his family, i.e. breast cancer in his mother and ovarian cancer in his sister, as well as Pt2 with breast cancer in his mother and prostate cancer in his father and Pt7 also with breast cancer in his mother.

## Theme 2: Dealing with the germline genetic testing result

We asked patients to measure how much of a psychological burden the genetic test result was on a scale from 0 (low) to 10 (high). Nearly all patients scored the psychological burden as 0 to 4, with two patients (Pt1, *BRCA2*; Pt3, *CHEK2*) scoring a 5. Despite the fact that several patients reported issues that could have negatively influenced their psychosocial wellbeing, no one indicated an interest in psychosocial support from a psychologist or a social worker to cope with the genetic test results. In Table 4, quotes about psychosocial consequences for the index patient are summarized. These quotes are either relevant for men with metastatic prostate cancer, or are interesting because they show a difference between high and moderate risk cancer genes. Multiple quotes were given regarding psychosocial consequences and therapy options. On one hand, a patient with a PV in *BRCA2* was hopeful due to having an extra treatment option [PARP inhibitors], while a patient with a PV in *CHEK2 *was disappointed because extra treatment options lacked. Only patients with a PV in a moderate risk cancer gene quoted that they were unimpressed by the genetic test result, while only patients with a PV in a high risk cancer gene said that they felt guilt about possibly having passed on the PV.

**Table 4:** Psychosocial consequences of genetic test results for the index patient

Consequences	Patient	Quote
• Reassurance	Pt2 (*CHEK2*)	*‘When I heard what the genetic predisposition meant for me and my relatives, I was actually reassured, because I believe it is not a genetic predisposition that is severe.’ … ‘It could also have been that everyone had a 100% chance of something.’*
• Contentment	Pt14 (*CHEK2*)	*‘Happy to see that there was no connection between this variant and the prostate cancer’*
• Hopeful	Pt1 (*BRCA2*)	*‘There was one more option in the treatment of prostate cancer’*
• Unimpressed	Pt2 (*CHEK2*), Pt8 (*ATM*), and Pt13 (*ATM*)	*‘You’re bit surprised by it, but it did not upset me’ /* *‘That genetic test was not emotionally stressful. When I got the message 9 years back that I had prostate cancer, I did have a hard time with that. But not the test.’ (Pt8)*
• Concerned	Pt1 (*BRCA2*), Pt2 (*CHEK2*), Pt4 (*BRCA2*), Pt7 (*ATM*) and Pt10 (*PALB2*)	*‘I knew what BRCA meant, because of course you go and apply for one of those genetic tests and then you know what could possibly come out… and then I am frightened especially for my sisters and daughters, because they could have it too.’ (Pt1)*
• Guilt	Pt1 (*BRCA2*) and Pt4 (*BRCA2*)	*‘But I might have felt guilty towards my children if I had passed it on’ (Pt4)*
• Disappointment	Pt3 (*CHEK2*)	*‘I hoped that it was [a variant] that gave extra access to a treatment. However, I got over that disappointment pretty quickly too.’*
• Frustration	Pt11 (*BRCA2*)	*‘The test result for me was too little too late and bad luck.’*

## Theme 3: Navigating family factors in disclosure of genetic testing results and recommendations for cascade testing

Three patients stated that they informed relatives because of possible preventive measures that could be taken (Pt1, *BRCA2*; Pt4, *BRCA2* and Pt8, *ATM*) or out of responsibility for their relatives (Pt1, *BRCA2*; Pt9, *ATM*; Pt13, *ATM*).

Family communication took place in various ways, e.g. in person (Pt2, *CHEK2*; Pt6, *ATM*; Pt12, *CHEK2*; Pt14, *CHEK2*), by phone (Pt1, *BRCA2*; Pt3, *CHEK2*; Pt5, *CHEK2*; Pt13, *ATM*), by e-mail (Pt1, *BRCA2*; Pt6, ATM*;* Pt12, *CHEK2*), by text messages (Pt9, *ATM*). Sometimes family communication was performed by others, for example siblings, 2nd/3rd-degree relatives or their partner (Pt3, *CHEK2*; Pt5, *CHEK2*; Pt6, *ATM*; Pt10, *PALB2*; Pt11, *BRCA2*; Pt12, *CHEK2*) or by the genetics department (Pt4, *BRCA2*) when there was no contact with the relatives. If and how family communication took place did not seem to be associated with the score on the IRI positive motivation subscale. Patients who, on one hand, overall scored low on the positive motivation subscale but, on the other hand, informed most/all of their relatives, had at least one positive motivation that was important to them (Pt1, *BRCA2*; Pt2, *CHEK2*; Pt8, *ATM*).

The patients interviewed felt the family letter was complete, clear and necessary in communicating the genetic test result with their relatives.

*‘I thought that [the family letter] was fine. I actually thought that one was very well drafted even. It contained enough information and my children thought so and so did my brother.’ (Pt4, BRCA2)*

No patients had negative experiences with the family letter. One patient (Pt9, *ATM*) did not use the family letter.

Besides the family letter, using e-mail was felt to be helpful in the family communication (Pt1, *BRCA2*) for handing out the family letter. Furthermore, it was helpful if there was good contact within the family. On the other hand, some patients had no or little contact with relatives, or relatives who lived abroad. Having little contact was not always a barrier to family communication, shown by a patient who felt so much responsibility towards his sister that he contacted her despite not having contact for over 40 years (Pt13, *ATM*). Relatives’ age (being too young or too old) interfered with family communication, especially when the elderly relative was childless (Pt8, *ATM*). Psychological and psychiatric diseases in relatives were listed as being challenging in family communication (Pt4 *BRCA2*; Pt6, *ATM*) and for Pt6 this was a reason to not communicate the genetic test result. Combinations of multiple challenging factors (e.g. psychological distress and high age) were also mentioned as reasons for not communicating the genetic test result to relatives.

‘*My sister can be quite panicky, and the letter clearly stated that those breast exams can be enough. But she’ll be seventy-eight soon, and I reckon she won't even be eligible for that anymore.’ (Pt7, ATM)*

Two patients (Pt1, *BRCA2* and Pt9, *ATM*) had negative experiences when communicating the genetic test result to a relative. Both of them were disappointed that their relatives (a niece and a daughter, respectively) did not share the same sense of urgency about genetic testing. One patient strongly recommended that his relatives should undergo genetic testing (Pt13, *ATM*). Other patients only mentioned having positive experiences with the family communication and deemed it not difficult or ‘not so bad’ (Pt2, *CHEK2*; Pt5, *CHEK2*; Pt6, *ATM*; Pt10, *PALB2*; Pt11, *BRCA2*).

Patients experienced support from their partners and relatives not only in requesting genetic testing and dealing with the test results but also in family communication (Pt1, Pt2, Pt4, Pt5, Pt8, Pt11, Pt12, Pt13, Pt14). While most interviewed patients experienced this support, it did not seem to play an important role (2.7/5) in motivations to communicate the test result.

Index patients mentioned that certain barriers among relatives made the relatives refrain from undergoing genetic testing.

*‘It (i.e. genetic testing) is too expensive’ (Pt1, BRCA2)*

*‘It (i.e. genetic testing) is hard, because I live in [city name]’ (somewhere on the other side of the country) (Pt9, ATM)*

Others refrained from uptake of genetic testing because of anxiety (Pt11, *BRCA2*) or because they did not have enough time (Pt9, *ATM*). One relative postponed genetic testing due to an upcoming mortgage (Pt4, *BRCA2*).

When comparing family communication in families with PVs in high-risk versus moderate-risk cancer genes, no major differences can be seen. In both situations, index patients often felt a responsibility to communicate with at-risk relatives. The only difference was that relatives were less often at risk in the families with a moderate risk variant and the family communication was therefore less open towards relatives, especially cousins. Four patients, having a PV in a moderate-risk cancer gene (Pt3, *CHEK2*; Pt7, *ATM*; Pt8, *ATM*; Pt9, *ATM*), scored low on the IRI self-efficacy question ‘e’ on whether they were sure that they could ‘explain what the importance of the information is for them [their relatives]’. Furthermore, two of these patients who were not advised to inform relatives (Pt7, *ATM*; Pt8, *ATM*) scored significantly lower than all the others on the IRI positive motivation and self-efficacy subscale. They also estimated in the interviews their psychological burden lowest from all interviewees.

## Theme 4: Evaluating and taking action after disclosure of testing results

Index patients indicated that the detection of a PV and the subsequent family communication had diverse consequences for relatives. They said that their relatives had told them that they were shocked (Pt11, *BRCA2*) or stressed because they could harbour the same PV (Pt10, *PALB2*), frightened because their questions could not be answered immediately (Pt5, *CHEK2*) or worried that they might have to undergo surgery and therefore they have said that their feelings of femininity decreased (Pt1, *BRCA2*). On the other hand, some relatives reacted positively to the genetic test result, because now they knew that they could do something about it (Pt2, *CHEK2*). These risk-reducing surgeries (mastectomy and oophorectomy) were already planned by female relatives who carried the PV from Pt1 (*BRCA2*). One of these relatives was no longer of childbearing age and therefore said to the index patient that she had fewer objections to oophorectomy than to a mastectomy. Measures were also undertaken by male relatives with periodic PSA screening. Male relatives of a *BRCA2* (Pt4) and *ATM* carrier (Pt9) already underwent periodic PSA screening before the genetic test result and insistent on continuing. Multiple relatives of a *CHEK2* carrier (Pt5) started PSA screening on their own initiative. These three index patients all scored high on the IRI positive subscale question ‘j’ ‘I would like to encourage my relative to go for regular screening’.

## Discussion

This mixed-methods study shows that metastatic prostate cancer patients with a PV in high-risk or moderate-risk breast cancer predisposition genes feel well-equipped to communicate about this PV in their families. Furthermore, patients feel very much responsible for informing relatives about the PV. We found that it was crucial to have at least one important motivator within family communication. Patients with a PV in a moderate risk cancer gene were more insecure in communicating the relevance of the test result with relatives.

Family communication is essential in cascade screening. Previous studies have shown that the uptake of genetic testing in hereditary cancer is low [44] and difficult to improve [45]. A limited family communication style is one factor that may contribute to the low uptake of genetic testing. Our patients overall felt well-equipped (high self-efficacy) for family communication, as well as having numerous motivations for communicating with their relatives. The mean score (40/65) of the IRI subscale ‘positive motivation’ was identical to the mean score of the original paper [40], indicating that our patients with metastatic prostate cancer were equally motivated to communicate with relatives as the cohorts of patients with hereditary breast/ovarian and colon cancer. The ‘negative motivation’ and ‘self-efficacy’ were also nearly identical with 29 versus 27 and 21 versus 20 respectively. However, mean scores should be interpreted with caution when using small sample sizes like in our study [46]. Patients who overall had a low score on the positive motivation subscale, still informed their relatives when they at least had a few motivations that were important to them. It is therefore crucial to find an important motivator for patients to encourage them to communicate the genetic test result with their relatives. Within the post-test counselling consultation a specific motivator for the index patient should be searched in order to increase the likelihood of open family communication and corresponding cascade screening. The 13 positive motivators in the IRI questionnaire could be a guidance towards this search. For future studies, it might be interesting to assess the actual uptake of genetic testing by relatives, when the PV in the family is detected first in the prostate cancer patient.

Patients in our study often felt the germline genetic test result to be less stressful than the cancer diagnosis, which was a confirmation of a previous study in prostate cancer patients [35]. As was also described previously, prostate cancer patients with a PV might feel guilt towards offspring due to possibly passing on the PV [35]. In our patient cohort, only patients with a *BRCA2* variant experienced this guilt. This may be caused by the fact that having a PV in moderate-risk breast cancer genes *CHEK2* and *ATM* does not always have direct consequences for relatives, which is in contrast to carriers of a PV in a high-risk gene like *BRCA2*. Four patients with a PV in a moderate-risk cancer gene experienced uncertainty towards explaining the importance of the test result to relatives (IRI self-efficacy question ‘e’). Two of these patients were advised to inform relatives, and therefore they should have been informed during a post-test counselling consultation about the importance. It is crucial that index patients transfer information on the importance of genetic testing, since uncertainty about the utility of genetic testing has proven to be a barrier towards the uptake of genetic testing in women [47]. However, index patients in the mentioned study were women with a personal or family history with breast cancer, who were eligible for multigene (n = 25) panel testing and their response might differ from women who have a relative that is proven carrier of a PV. Nonetheless, in a post-test counselling consultation it should be checked whether the index patient understood the utility of genetic testing for relatives. Fear for relatives was experienced by carriers of PVs in each gene, while the cancer risks between these genes can be significantly different for relatives who carry the same PV. Overall, patients said that the psychological burden of the genetic test result was fairly low.

One factor that significantly limited family communication was having little or no contact with relatives, while these relatives can carry the same PV. If this is the case, genetics departments might help patients contact these relatives. Some patients had a limited or closed family communication style when they deemed the information not useful for their relatives. It might be possible that patients decided that themselves, or that their clinical geneticist explained which relatives were eligible for genetic testing. Clinical geneticists should be aware of patients who decide for themselves that information is not useful for anyone, and determine if this is truly the case. In addition, several barriers towards germline genetic testing were being reported by relatives that withheld them from informing relatives. Barriers such as ‘genetic testing is expensive’ should be countered by more nationwide public education, because genetic testing in the Netherlands is fully paid for by health insurance companies [48]. Only, annually there is an own contribution of at least 385 euros for a person’s total healthcare costs. Barriers that made relatives refrain from the uptake of genetic testing were present both in families with a PV in high-risk and moderate-risk cancer genes. In family communication, no major differences were identified between families with a PV in high-risk or moderate-risk cancer genes, despite family communication being less extensive towards cousins in families with a PV in a moderate-risk gene. In these families, there is less often a reason to perform genetic testing in cousins and family communication about the genetic testing result is therefore less urgent.

Several carriers of a PV in a high risk cancer gene reported that the disclosure of the test result impacted their relative and that their relatives were shocked, stressed or worried because of possible surgery. It shows that these patients were able to not only disclose the genetic test result, but also disclose the relevance of it. Some patients had a male relative who did not opt for germline genetic testing, but had PSA screening done instead. This was for example the case in relatives of PV carriers in *ATM* and *CHEK2*. For these genes, there is currently no guideline that recommends PSA screening in carriers of a PV. There are also no supporting guidelines yet for men who are at risk and are not proven PV carriers.

The reasons for men with metastatic prostate cancer to undergo germline genetic testing are numerous. As known from prior studies and confirmed in ours, men participate in genetic testing to get a better picture of the cancer risks for relatives, to take possible preventive measures or out of curiosity [8, 35]. However, men in our study often underwent genetic testing to determine if they had a PV that may lead to an extra treatment option. Some men even hoped to have a PV, not fully realizing that their relatives could harbour the same PV and therefore be at an increased risk of cancer. Men are often unaware of these cancer risks [35]. When men receive pre-test counselling, healthcare professionals should emphasize the relatives’ cancer risks, especially in a patient group of men with advanced cancer whose treatment depends (more often than patients with localized disease) on the genetic test result.

Interestingly, three patients were interviewed who did not expect to have a PV, despite their families were suffering from relevant cancer types, like ovarian and breast cancer. This shows that patients do not widely know the existing link between prostate, ovarian, breast and other cancers. Nationwide education should be encouraged to ensure that healthcare professionals provide the full range of consequences (including the link between prostate cancer and other cancers) and to create awareness around informing patients about this.

A limitation of this study are the relatively small number of patients who consented to an interview. Only 14 out of 23 (61%) carriers consented. Despite the limited interview compliance, it is much higher than a previous interview study in men with prostate cancer, which had a compliance rate of 19% [35]. The group who consented were slightly younger (aged 67 versus 75) and their educational level was significantly higher. People with a higher educational level often also have higher health literacy (the ability to understand and use health-related information), which probably makes transferring the correct medical information easier [49, 50]. A higher educational level in a subject has been shown to lead to higher uptake of genetic testing in relatives [51]. This could lead to inequality in health and lifespan in relatives when an index patient has a lower educational level. This emphasizes the need for more education on genetics to increase knowledge on genetics, also for those who have lower educational levels. The language and terminology used in this education should be tailored to people with lower education levels to make up for this inequality. Future studies should be done to assess family communication in patients with metastatic prostate cancer and lower health literacy. Another limitation is that we do not know whether open family communication will automatically result in a higher uptake of genetic testing among relatives. Future studies can be done to assess this.

Furthermore, although the genes in our study are all moderate to high-risk cancer genes, there still were four men with a PV in *ATM* or *CHEK2*, who were not advised to inform relatives about the PV, for example because all the relatives were elderly or because there were no breast cancer cases in the family. Having a breast cancer case in families with an *ATM* or *CHEK2* variant is a prerequisite in the Netherlands for performing genetic testing in relatives, as it would otherwise not lead to any preventive options in relatives. This advice may change over the years; some patients were therefore given the advice to inform relatives, while this might not be the case a few years later, and vice versa.

## Conclusion

Patients with metastatic prostate cancer and a hereditary predisposition to cancer feel well-equipped to communicate about this predisposition in their family. Patients felt a strong sense of obligation and responsibility towards their relatives. In patients with low motivation to pursue family communication, effort should be made to find at least one important motivator.

### Supplementary Information

Below is the link to the electronic supplementary material.

Supplementary file1 (DOCX 19 KB)

Supplementary file2 (DOCX 13 KB)

Supplementary file3 (PDF 419 KB)

## Data Availability

The data are not publicly available due to privacy or ethical restrictions.

## References

[1] Sung H (2021). Global cancer statistics 2020: GLOBOCAN estimates of incidence and mortality worldwide for 36 cancers in 185 countries. CA Cancer J Clin.

[2] Buzzoni C (2015). Metastatic prostate cancer incidence and prostate-specific antigen testing: new insights from the European randomized study of screening for prostate cancer. Eur Urol.

[3] Hamdy FC (2016). 10-year outcomes after monitoring, surgery, or radiotherapy for localized prostate cancer. N Engl J Med.

[4] Abdi B (2022). DNA damage repair gene germline profiling for metastatic prostate cancer patients of different ancestries. Prostate.

[5] Abida W (2017). Prospective genomic profiling of prostate cancer across disease states reveals germline and somatic alterations that may affect clinical decision making. JCO Precis Oncol.

[6] Boyle JL (2020). Pathogenic germline DNA repair gene and HOXB13 mutations in men with metastatic prostate cancer. JCO Precis Oncol.

[7] Castro E (2019). PROREPAIR-B: a prospective cohort study of the impact of germline DNA repair mutations on the outcomes of patients with metastatic castration-resistant prostate cancer. J Clin Oncol.

[8] Giri VN (2017). Inherited mutations in men undergoing multigene panel testing for prostate cancer: emerging implications for personalized prostate cancer genetic evaluation. JCO Precis Oncol.

[9] Greenberg SE (2021). Clinical germline testing results of men with prostate cancer: patient-level factors and implications of NCCN guideline expansion. JCO Precis Oncol.

[10] Hart SN (2016). Determining the frequency of pathogenic germline variants from exome sequencing in patients with castrate-resistant prostate cancer. BMJ Open.

[11] Isaacsson Velho P (2018). Intraductal/ductal histology and lymphovascular invasion are associated with germline DNA-repair gene mutations in prostate cancer. Prostate.

[12] Na R (2017). Germline mutations in ATM and BRCA1/2 distinguish risk for lethal and indolent prostate cancer and are associated with early age at death. Eur Urol.

[13] Nguyen-Dumont T (2021). Rare germline pathogenic variants identified by multigene panel testing and the risk of aggressive prostate cancer. Cancers.

[14] Nicolosi P (2019). Prevalence of germline variants in prostate cancer and implications for current genetic testing guidelines. JAMA Oncol.

[15] Petrovics G (2019). Increased frequency of germline BRCA2 mutations associates with prostate cancer metastasis in a racially diverse patient population. Prostate Cancer Prostatic Dis.

[16] Priestley P (2019). Pan-cancer whole-genome analyses of metastatic solid tumours. Nature.

[17] Pritchard CC (2016). Inherited DNA-repair gene mutations in men with metastatic prostate cancer. N Engl J Med.

[18] Robinson D (2015). Integrative clinical genomics of advanced prostate cancer. Cell.

[19] Yadav S (2019). Contribution of inherited DNA-repair gene mutations to hormone-sensitive and castrate-resistant metastatic prostate cancer and implications for clinical outcome. JCO Precis Oncol.

[20] Bychkovsky BL (2022). Differences in cancer phenotypes among frequent CHEK2 Variants and implications for clinical care—checking CHEK2. JAMA Oncol.

[21] Hall MJ (2021). Germline pathogenic variants in the ataxia telangiectasia mutated (ATM) gene are associated with high and moderate risks for multiple cancers germline ATM PVs are associated with multiple cancer risks. Cancer Prev Res.

[22] Li S (2022). Cancer risks associated with BRCA1 and BRCA2 pathogenic variants. J Clin Oncol.

[23] Yang X (2020). Cancer risks associated with germline PALB2 pathogenic variants: an international study of 524 families. J Clin Oncol.

[24] Campos FAB (2021). Genetic landscape of male breast cancer. Cancers.

[25] Carbine NE (2018). Risk-reducing mastectomy for the prevention of primary breast cancer. Cochrane Database Syst Rev.

[26] Ludwig KK (2016). Risk reduction and survival benefit of prophylactic surgery in BRCA mutation carriers, a systematic review. The American Journal of Surgery.

[27] Wood ME, McKinnon W, Garber J (2020). Risk for breast cancer and management of unaffected individuals with non-BRCA hereditary breast cancer. Breast J.

[28] Page EC (2019). Interim results from the IMPACT study: evidence for prostate-specific antigen screening in BRCA2 mutation carriers. Eur Urol.

[29] de Bono JS (2021). Talazoparib monotherapy in metastatic castration-resistant prostate cancer with DNA repair alterations (TALAPRO-1): an open-label, phase 2 trial. Lancet Oncol.

[30] Fizazi K (2023). Rucaparib or physician’s choice in metastatic prostate cancer. N Engl J Med.

[31] Hussain M (2020). Survival with olaparib in metastatic castration-resistant prostate cancer. N Engl J Med.

[32] Dutch Clinical Genetics Society (2019) Richtlijn Informeren van familieleden bij erfelijke aandoeningen. Retrieved from https://www.vkgn.org/files/5911/Richtlijn%20informeren%20van%20familieleden%20bij%20erfelijke%20aandoeningen.pdf.

[33] Dheensa S, Lucassen A, Fenwick A (2018). Limitations and pitfalls of using family letters to communicate genetic risk: a qualitative study with patients and healthcare professionals. J Genet Couns.

[34] Finn CM (2023). Motivation and family communication in hereditary prostate cancer genetic testing: survey of patients from a US tertiary medical center. J Genet Couns.

[35] Leader AE (2022). Insight into how patients with prostate cancer interpret and communicate genetic test results: implications for families. J Community Genet.

[36] Vlaming M (2022). Mainstream germline genetic testing in men with metastatic prostate cancer: design and protocol for a multicenter observational study. BMC Cancer.

[37] Kroll T, Neri M (2009). Designs for mixed methods research. Mixed methods research for nursing and the health sciences.

[38] Kuper A, Lingard L, Levinson W (2008). Critically appraising qualitative research. BMJ.

[39] Standaard Onderwijsindeling (2021) Available from: https://www.cbs.nl/nl-nl/onze-diensten/methoden/classificaties/onderwijs-en-beroepen/standaard-onderwijsindeling--soi--/standaard-onderwijsindeling-2021.

[40] de Geus E (2015). Development of the informing relatives inventory (IRI): assessing index patients’ knowledge, motivation and self-efficacy regarding the disclosure of hereditary cancer risk information to relatives. Int J Behav Med.

[41] Claes E (2003). Communication with close and distant relatives in the context of genetic testing for hereditary breast and ovarian cancer in cancer patients. Am J Med Genet A.

[42] Braun V, Clarke V (2006). Using thematic analysis in psychology. Qual Res Psychol.

[43] Tong A, Sainsbury P, Craig J (2007). Consolidated criteria for reporting qualitative research (COREQ): a 32-item checklist for interviews and focus groups. Int J Qual Health Care.

[44] Menko FH (2019). The uptake of presymptomatic genetic testing in hereditary breast-ovarian cancer and Lynch syndrome: a systematic review of the literature and implications for clinical practice. Fam Cancer.

[45] Menko FH (2023). Does a proactive procedure lead to a higher uptake of predictive testing in families with a pathogenic BRCA1/BRCA2 variant? A family cancer clinic evaluation. J Genet Couns.

[46] Piovesana A, Senior G (2018). How small is big: sample size and skewness. Assessment.

[47] Bradbury AR (2016). Patient feedback and early outcome data with a novel tiered-binned model for multiplex breast cancer susceptibility testing. Genet Med.

[48] Kroneman M (2016). Netherlands: health system review. Health Syst Transit.

[49] Van Der Heide I (2013). The relationship between health, education, and health literacy: results from the Dutch adult literacy and life skills survey. J Health Commun.

[50] Harrison C (2023). Family communication and results disclosure after germline sequencing: a mixed methods study. Patient Educ Couns.

[51] Sanz J (2010). Uptake of predictive testing among relatives of BRCA1 and BRCA2 families: a multicenter study in northeastern Spain. Fam Cancer.

