# Supplementary material for: Men with metastatic prostate cancer carrying a pathogenic germline variant in breast cancer genes: disclosure of genetic test results to relatives
Source: Fam Cancer. 2024 May 9;23(2):165–75. doi: 10.1007/s10689-024-00377-0 (PMC11153271; doi:10.1007/s10689-024-00377-0)
Supplement: Supplementary file 1 — Supplementary file1 (DOCX 19 KB) [file 10689_2024_377_MOESM1_ESM.docx]

**Table S1: Detailed score per question on IRI questionnaire**

| **The motivation plays … (number of participants)** | | | | | | | |
| --- | --- | --- | --- | --- | --- | --- | --- |
|  | **1 – no role** | **2 – a minor role** | **3 – a reasonable role** | | **4 – a big role** | | **5 – a large role** |
| **Positive motivation** | | | | | | | |
| a. I feel obliged to provide information | 1 (4%) | 1 (4%) | 2 (9%) | | 10 (44%) | | 9 (39%) |
| b. I was encouraged by the health professional | 9 (39%) | 7 (30%) | 4 (17%) | | 0 (0%) | | 3 (13%) |
| c. I already promised my relatives to inform them before I got the result | 6 (26%) | 3 (13%) | 3 (13%) | | 9 (39%) | | 2 (9%) |
| d. I’m encouraged by other relatives to disclose the information | 11 (48%) | 3 (13%) | 4 (17%) | | 3 (13%) | | 2 (9%) |
| e. I think the information can help them to make medical decisions | 2 (9%) | 0 (0%) | 3 (13%) | | 10 (44%) | | 8 (35%) |
| f. A relative asked me about the test result | 10 (44%) | 2 (9%) | 6 (26%) | | 3 (13%) | | 2 (9%) |
| g. I would like to have my relatives’ advice when I make medical decisions | 11 (48%) | 3 (13%) | 6 (26%) | | 3 (13%) | | 0 (0%) |
| h. I need emotional support from my family | 6 (26%) | 4 (17%) | 4 (17%) | | 8 (35%) | | 1 (4%) |
| i. I would like to encourage my relative to go for genetic services | 5 (22%) | 1 (4%) | 7 (30%) | | 7 (30%) | | 3 (13%) |
| j. I would like to encourage my relative to go for regular screening | 4 (17%) | 1 (4%) | 6 (26%) | | 8 (35%) | | 4 (17%) |
| k. I understand the test result well | 0 (0%) | 1 (4%) | 5 (22%) | | 11 (48%) | | 6 (26%) |
| l. I have a close relationship with some of my relatives | 1 (4%) | 0 (0%) | 3 (13%) | | 11 (48%) | | 8 (35%) |
| m. I think relatives’ children should know the information | 1 (4%) | 2 (9%) | 3 (13%) | | 7 (30%) | | 10 (44%) |
| **Negative motivation*** | | | | | | | |
| a. I do not have any contact with some of my relatives | 3 (33%) | 1 (11%) | 2 (22%) | | 1 (11%) | | 2 (22%) |
| b. I do not have a good relationship with some of my relatives** | 4 (50%) | 1 (13%) | 1 (13%) | | 2 (25%) | | 0 (0%) |
| c. I do not believe the information would be useful to them | 4 (44%) | 0 (0%) | 3 (33%) | | 2 (22%) | | 0 (0%) |
| d. I do not think the relative can handle the information emotionally | 5 (56%) | 2 (22%) | 1 (11%) | | 1 (11%) | | 0 (0%) |
| e. I am worried about being responsible for causing problems in a relationship or marriage | 8 (89%) | 1 (11%) | 0 (0%) | | 0 (0%) | | 0 (0%) |
| f. My relative told me they did not want to know | 7 (78%) | 2 (22%) | 0 (0%) | | 0 (0%) | | 0 (0%) |
| g. I do not have time to tell my relatives | 7 (78%) | 2 (22%) | 0 (0%) | | 0 (0%) | | 0 (0%) |
| h. It is difficult to reach some of my relatives | 3 (33%) | 3 (33%) | 1 (11%) | | 1 (11%) | | 1 (11%) |
| i. I do not want to burden my relative, since he/she is having difficulties | 6 (67%) | 3 (33%) | 0 (0%) | | 0 (0%) | | 0 (0%) |
| j. I consider my relative too young to inform | 5 (56%) | 3 (33%) | 0 (0%) | | 0 (0%) | | 1 (11%) |
| k. It is emotionally difficult for me to share the information | 5 (56%) | 3 (33%) | 1 (11%) | | 0 (0%) | | 0 (0%) |
| l. The information is so complex, I do not know how to share the information | 6 (67%) | 3 (33%) | 0 (0%) | | 0 (0%) | | 0 (0%) |
| m. I do not know who has an increased risk for having hereditary cancer | 6 (67%) | 3 (33%) | 0 (0%) | | 0 (0%) | | 0 (0%) |
| n. Some relatives do not understand how the information applies to them | 5 (56%) | 2 (22%) | 2 (22%) | | 0 (0%) | | 0 (0%) |
| o. I do not want to upset my relatives | 3 (33%) | 2 (22%) | 2 (22%) | | 2 (22%) | | 0 (0%) |
| p. I feel guilty or anxious about the test result | 6 (67%) | 3 (33%) | 0 (0%) | | 0 (0%) | | 0 (0%) |
| q. I feel the information is too personal to share | 7 (78%) | 2 (22%) | 0 (0%) | | 0 (0%) | | 0 (0%) |
| **(number of participants)** | | | | | | | |
|  | **1 – not sure at all** | **2 – somewhat sure** | | **3 – sure** | | **4 – very sure** | |
| **Self-efficacy** | | | | | | | |
| *If you would like to inform your family, how sure are you that you …*  a. Find the time to speak them | 0 (0%) | 1 (4%) | | 12 (52%) | | 10 (44%) | |
| b. Contact them | 1 (4%) | 1 (4%) | | 11 (48%) | | 10 (44%) | |
| c. Disclose the information clearly | 1 (4%) | 3 (13%) | | 11 (48%) | | 8 (35%) | |
| d. Cause undesirable turbulence | 4 (17%) | 7 (30%) | | 8 (35%) | | 4 (17%) | |
| e. Explain what the importance of the information is for them | 1 (4%) | 4 (17%) | | 13 (57%) | | 5 (22%) | |
| f. Avoid problems in the relationship | 1 (4%) | 3 (13%) | | 13 (57%) | | 6 (26%) | |
| g. Have enough knowledge | 2 (9%) | 5 (22%) | | 11 (48%) | | 5 (22%) | |

* n = 9

** n = 8
