# Supplementary material for: Men with metastatic prostate cancer carrying a pathogenic germline variant in breast cancer genes: disclosure of genetic test results to relatives
Source: Fam Cancer. 2024 May 9;23(2):165–75. doi: 10.1007/s10689-024-00377-0 (PMC11153271; doi:10.1007/s10689-024-00377-0)
Supplement: Supplementary file 2 — Supplementary file2 (DOCX 13 KB) [file 10689_2024_377_MOESM2_ESM.docx]

**Table S2: Mean scores for the subscales of the IRI questionnaire (n = 23)**

| **Subscale** | **Total group*,**  **Mean (SD)** | **Reference article (n = 204)** [35],**  **Mean (SD)** |
| --- | --- | --- |
| Positive motivation (min: 13 – max: 65) | 41 (10) | 40 (14) |
| Negative motivation (min: 17 – max: 85) | 29 (4) | 27 (13) |
| Self-efficacy (min: 7 – max: 28) | 21 (5) | 20 (6) |
| * n = 9 for negative motivations  ** n = 71 for negative motivations |  |  |
